# Medical Treatment during the Adolescent Period

**Published:** 1901-06

**Authors:** 


### Abstract.

### MEDICAL TREATMENT DURING THE ADOLESCENT PERIOD.

THE adolescent period in the female, says Edwin Rosenthal, Philadelphia (*The Medical Fortnightly*), may be said to be as critical in results as the menopause, and by reason of the methods of our education may be said to be one of the best known conditions universally recognised by the profession and the laity.

Two classes of cases are most numerous: (1) those who have never menstruated, and (2) that class that may have begun, shown a very slight discharge at infrequent intervals, but which has never grown to an extent at any time that may be termed a normal flow.

In all cases of menstrual disorders in the young, the cause must be sought for, and if found corrected. This of certainty directs the treatment. In cases where the menstruation has never appeared, it should always be a certain rule to have the sufferer examined by the mother. In quite a number of instances, anatomical reasons have shown the cause. In four cases an impervious hymen was the cause. In two cases the uterus became the receptacle, and contained the result of numerous menstruations, becoming enlarged even above the pubic bones; the cervix being impervious. In several instances there was an entire absence of the uterus and ovaries. In one case there was an absence of a vagina. Such cases are thus enumerated; nothing can be done in the line of medication, but judicious surgical procedures may in indicated cases effect a cure. Where, however, no anatomical reasons exist, and the patient suffers from suppression of the menstruation, entire or in part, much can be done to aid a cure.

The most common symptom of disordered menstruation is anemia or chlorosis. Anemia alone may be the cause of suppressed menstruation, and while its presence may be looked upon as a certain cause, its treatment is as essential for the appearance of the menstruation as it should be for the general health of the patient.

Besides the condition of the blood as a cause of suppressed menstruation, other well-known conditions equally play a prominent part. Even if the patient should suffer from such diseases—tuberculosis as an example—the presence of a menstrual flow has such an encouraging influence upon the mind of the sufferer, that some attempt should be made to establish it.

Iron is the chief remedy in menstrual disorders, and may be given at all times—before, after and during the flow.

Between the periods I always order the use of iron in three or four daily doses. I have used all forms and varieties, from the tincture of the chloride, which is so often objected to, to the different kinds the pharmacopeia presents, in pill form, as the Blaud pill, simple or modified. My experience brings me back to Gude's pepto-mangan. Gude's pepto-mangan is now the most common in use, and there are so very many similar preparations in the apothecaries that care should be exercised in obtaining the genuine. I have a simple way of distinction. I always order Gude's pepto-mangan given with milk. If the mixture is clean, uncoagulated and palatable, then I know my patient has received what I ordered. For further distinction, I invariably place on my prescription the name "Gude." I order this preparation a teaspoonful in a wineglassful of milk every three or four hours, depending upon the patient's condition.

I have seen such good results in the use of Gude's pepto-mangan in septic diseases, that I have applied it fearlessly in other conditions. None give better promise than those conditions that are coupled with the menstrual flow, especially when seen at the adolescent period.

---

Wearing of Belts after Abdominal Section.—Patients who have undergone abdominal section should wear a well-fitting belt for at least two years after the operation, otherwise a hernia is very apt to form. I am aware that more than one distinguished abdominal surgeon has denounced the belt as tending to cause atrophy of the abdominal muscles. Personally, I do not think for one moment that it does; but even if it were so, a somewhat weak abdominal wall is better than a ventral hernia.—Christopher Martin, in *The Scalpel.*

